# Induction of hepatic fibrosis in mice with schistosomiasis by extracellular microRNA-30 derived from *Schistosoma japonicum* eggs

**DOI:** 10.3389/fimmu.2024.1425384

**Published:** 2024-07-30

**Authors:** Yang Chen, Yuan Hu, Hao Zhou, Nan Jiang, Yiluo Wang, Jing Zhang, Yujuan Shen, Guoying Yu, Jianping Cao

**Affiliations:** ^1^ State Key Laboratory of Cell Differentiation and Regulation, College of Life Science, Pingyuan Laboratory, Henan Normal University, Xinxiang, China; ^2^ National Key Laboratory of Intelligent Tracking and Forecasting for Infectious Diseases, National Institute of Parasitic Diseases at Chinese Center for Disease Control and Prevention, Chinese Center for Tropical Diseases Research, Shanghai, China; ^3^ Key Laboratory of Parasite and Vector Biology, National Health Commission of the People’s Republic of China, Shanghai, China; ^4^ World Health Organization Collaborating Centre for Tropical Diseases, Shanghai, China; ^5^ The School of Global Health, Chinese Center for Tropical Diseases Research, Shanghai Jiao Tong University School of Medicine, Shanghai, China

**Keywords:** schistosomiasis, *Schistosoma japonicum*, exosomes, microRNA-30, hepatic fibrosis

## Abstract

**Background:**

Schistosomiasis is a zoonotic parasitic disorder induced by the infestation of schistosomes, a genus of trematodes. MicroRNAs (miRNAs) in egg-derived exosomes are crucial for modulating the host’s immune responses and orchestrating the pathophysiological mechanisms. Although the exosomes secreted by *S. japonicum* contain abundant miRNAs, the specific roles of these miRNAs in the pathogenesis of schistosomiasis-induced hepatic fibrosis are yet to be comprehensively elucidated. The egg exosomes of *S. japonicum* secrete miRNA-30, a novel miRNA.

**Methods:**

*In vitro*, the effect of miRNA-30 was evaluated by transfecting HSCs with miRNA mimics. The target gene biosignature for miRNA-30 was predicted using the miRDB software. The effect of miRNA-30 in hepatic fibrosis was evaluated by either elevating its expression in healthy mice or by inhibiting its activity in infected mice by administration of recombinant adeno-associated virus serotype eight vectors expressing miRNA-30 or miRNA sponges.

**Results:**

This novel miRNA can activate hepatic stellate cells (HSCs), the prinary effector cells of hepatic fibrosis, *in vitro*, i.e., it significantly increases the fibrogenic factors *Col1*(*α1*), *Col3*(*α1*), and *α-SMA* at both mRNA and protein levels. In addition, miRNA-30 may activate HSCs by targeting the host RORA gene. In addition, *in vivo* experiments were conducted by administering a recombinant adeno-associated viral vector to modulate the expression levels of miRNA-30. The overexpression of miRNA-30 in healthy mice significantly elevated the expression of *Col1*(*α1*), *Col3*(*α1*), and *α*-SMA at both the transcriptomic and proteomic scales. This overexpression was coupled with a pronounced augmentation in the hepatic hydroxyproline content. Conversely, the *in vivo* silencing of miRNA-30 in infected mice induced a considerable reduction in the size of hepatic granulomas and areas of collagen deposition. Hence, *in vivo*, modulation of miRNA-30 expression may play a pivotal role in ameliorating the severity of hepatic fibrosis in mice afflicted with *S. japonica*.

**Conclusions:**

The study results suggest that miRNA-30 may augment schistosomiasis-induced hepatic fibrosis through a probable interaction with the host RORA. Our study may improve the current theoretical framework regarding cross-species regulation by miRNAs of hepatic fibrosis in schistosomiasis.

## Introduction

Schistosomiasis, a zoonotic infection, is caused by the parasitic flatworm *Schistosoma* spp (1). It has been reported from across 78 tropical and subtropical regions (2). It is a neglected tropical disease, even though it impacts an estimated 230 million people globally (3). The parasite lays its eggs within host tissues or the portal venous system, which triggers various immunopathological reactions in the host, such as the inflammation and blockage of the urinary system, multiple digestive issues such as diarrhea and bloody stools, as well as the inflammation of the liver and spleen, including liver fibrogenesis (notably in infections by *Schistosoma mansoni* and *S. japonicum*) (4, 5). The *S. japonicum* is reported to be historically pervasive in China. Although China has made substantial progress in the mitigation of schistosomiasis over the past decade, 112 counties (inclusive of cities and districts) of the country are still affected by the parasite. Presently, schistosomiasis transmission is primarily managed by using praziquantel (PZQ), an anthelmintic that is mainly effective against adult schistosomal parasites. It is ineffective against other developmental stages of the parasite (6). Therefore, the therapeutic resolution of schistosomiasis remains a formidable global health challenge (7).

Hepatic fibrosis is characterized by fibrous scars produced through the aggregation of extracellular matrix (ECM) proteins, which predominantly comprise cross-linked collagen types I and III. In a fibrotic liver, these fibrous scars substitute the damaged normal hepatic tissue (8, 9). Myofibroblasts are the principal components of the ECM in fibrotic livers. Hence, mitigating efforts for fibrosis must target myofibroblasts (10, 11). *S. japonicum* larvae infect their human hosts by direct penetration through the skin. Once the larvae reach the adult stage, they lay eggs, eliciting an intensified immune response in the host against the soluble egg antigen (SEA) (12, 13). This intense immune reaction induces the proliferation of hepatic stellate cells (HSCs), which subsequently transform into myofibroblasts (9, 14). This conversion is accompanied by an extensive synthesis of ECM, resulting in secondary hepatic fibrosis and cirrhosis (15, 16). Upon stimulation by the SEA, HSCs up-regulate the expression of *α-SMA*. Activated HSCs migrate to the injury site and secrete ECM, resulting in fibrous scarring (17, 18). Several cytokines directly stimulate *Col1*(*α1*) transcription in activated HSCs. Transforming growth factor-beta (TGF-β) is the most potent pro-fibrotic cytokine and drives smad2/smad3-dependent HSC activation (18, 19).

MicroRNAs (miRNAs) are short endogenous non-coding RNAs (18–25 nucleotides) that modulate post-transcriptional gene expression by binding to the 3′-untranslated region (3′-UTR) or open reading frame of target gene mRNAs (20, 21). Note that miRNAs are involved in a myriad of biological processes, including cell proliferation, differentiation, migration, and disease initiation and progression (21–25). They are attracting increasing research interest because of their role in extracellular vesicles, particularly exosomes, which are critical to intercellular communication (26, 27). miRNAs delicately regulate gene expression involved in fibrogenesis (28). Recent investigations have highlighted the critical role of host miRNAs in schistosomiasis-induced hepatic fibrosis (29, 30). During the course of the disease, *S. japonicum* continues to secrete exosomes that carry numerous worm-derived miRNAs (31, 32). These exosomes, upon release, deliver the worm-derived miRNAs to host cells, thereby influencing host immunity and pathology (32).

We have conducted the sequencing of *S. japonicum* egg exosomes in a previous study. We have also reported various novel miRNAs potentially involved in the regulation of hepatic fibrosis in the host. Building upon the previous report, we herein identify miRNA-30 as a particularly promising candidate for further investigation.

## Materials and methods

### Ethics statement

All animal experiments were performed in strict accordance with the Regulations for the Administration of Affairs Concerning Experimental Animals (approved by the State Council of the People’s Republic of China). Efforts were made to minimize suffering. The study was approved by the Laboratory Animal Welfare & Ethics Committee (LAWEC) of the National Institute of Parasitic Diseases, Chinese Center for Disease Control and Prevention (Chinese Center for Tropical Diseases Research; approval ID: IPD 2019–12).

### Cell culture and treatment

Human HSCs of the LX-2 line were acquired from Wuhan Servicebio Technology Co. Ltd. (China). Mouse HSCs (mHSCs) were procured from Zhejiang Meisen Cell Technology Co. Ltd. (China). For the propagation of LX-2 cells, a high-sugar Dulbecco’s Modified Eagle Medium (DMEM, 11995073, Gibco, USA) augmented with 10% fetal bovine serum (FBS, 10091148, Gibco, USA) and 1% penicillin-streptomycin (PS, 15070063, Gibco, USA) was employed. The mHSCs were cultivated in commercially obtained Dulbecco’s/F-12 medium (D/F-12, CTCC-002-035, Meisen, China). Both LX-2 and mHSCs were maintained under physiological conditions conducive to cellular growth, specifically within an incubator at 37°C and in an atmosphere comprising 5% CO_2_.

A novel mimic of miRNA-30, synthesized by RiboBio (Guangzhou, China), was utilized for the transfection of both LX-2 and mHSC lines. The transfection was initiated when 30–50% of cellular confluence was achieved. Manufacturer-provided protocols were adhered to during the transfection process, wherein the cells were exposed to either a negative control (NC) mimic or miR-30 mimic at concentrations determined optimal for each cell type (LX-2: 100 and 150 nM; mHSC: 50 nM) and subsequently cultured for 48 h. The detailed miRNA sequences and primer sequences are shown in **Table 1**.

**Table 1:** miRNA sequences and primer sequences.

Gene	Primer sequences
U6 (human/mouse) sequences	GTGCTCGCTTCGGCAGCACATATA CTAAAATTGGAACGATACAGAGAA GATTAGCATGGCCCCTGCGCAAGG ATGACACGCAAATTCGTGAAGCGT TCCATATTTTT
U6 reverse (for RT PCR)	GTCGTATCCAGTGCAGGGTCCGAG GTATTCGCACTGGATACGACAAAA ATATGG
U6 qPCR-F	GCTCGCTTCGGCAGCACATATAC
U6 qPCR-R	AGTGCAGGGTCCGAGGTATT
Novel miRNA-30 sequences	AAGAGAGGCUGUAUUGAACC
Novel miRNA-30 reverse (for RT PCR)	GTCGTATCCAGTGCAGGGTCCGAG GTATTCGCACTGGATACGACGGTT CA
Novel-miRNA-30-qPCR-F	GCGCGAAGAGAGGCTGTAT
Novel-miRNA-30-qPCR-R	AGTGCAGGGTCCGAGGTATT

### Animals and parasite infections

All experimental animals were purchased from Shanghai Jihui Laboratory Animal Care Co., Ltd. (China) and were raised under specific pathogen-free conditions. All animal surgeries were performed under pentobarbital sodium-induced anesthesia to minimize pain and distress. The *S. japonicum* cercariae were supplied by the Vector Unit of the National Institute of Parasitic Diseases, which is part of the Chinese Center for Disease Control and Prevention under the National Research Centre for Tropical Diseases.

For the mass acquisition of *S. japonicum* eggs, three female New Zealand white rabbits were percutaneously exposed to 800 ± 20 *S. japonicum* cercariae. The rabbits were raised for 6 weeks and subsequently livers were harvested for the isolation of *S. japonicum* eggs. Fresh eggs were cultured for 1 week, the supernatant was collected every day, and the exosomes were separated by ultracentrifugation.

Fifty-four 6-week-old female C57BL/6 mice were randomly and equally divided into three groups. These mice were injected with 150 μL of PBS, rAAV8-SCR (blank vector), and rAAV8-miR-30 (overexpression vector) into the tail vein, with a viral titer of 1×10^12^ vector genomes per mouse. Then, on days 42, 56, and 70 post-injection, the mice were euthanized and liver samples were collected.

Fifty-four 6-week-old female C57BL/6 mice were randomly and equally allocated into three distinct groups and injected, via the tail vein, with 150 µL of PBS, rAAV8-SCR (blank vector), or rAAV8-anti-miR-30 (silencing vector), each at a viral load of 1×10^12^ vector genomes per mouse. At three weeks post-injection, the mice were percutaneously exposed to 20 ± 1 *S. japonicum* cercariae. On days 42 and 56 post-infection, these mice were euthanized, and the liver samples were extracted.

### rAAV8 design and production

This study employed rAAV8-SCR, rAAV8-miR-30, and rAAV8-anti-miR-30 that were designed, generated, purified, and titered by Vigene Biosciences (Shandong, China).

### Dual-luciferase reporter gene assay

The target gene biosignature for miRNA-30 was predicted using the miRDB software. Wild-type (WT) and 3′-UTR mutant reporter vectors for the *NT5E* and *RORA* genes were synthesized, and the dual-luciferase assay was executed.

### rAAV8 design and production

This study employed rAAV8-SCR, rAAV8-miR-30, and rAAV8-anti-miR-30 that were designed, generated, purified, and titered by Vigene Biosciences (Shandong, China).

### Western blot

Cellular and tissue specimens were lysed using the RIPA buffer with protease inhibitors. The protein concentrations were determined using the BCA Protein Assay Kit (PC0020, Solarbio, China) after conducting a thorough cell disruption. The samples were then heated for 10 min with 6 × Protein Loading Buffer. Next, 20–30 μg of protein was added per lane and the proteins were separated on SurePAGE™ (M00657, Genscript, China). After their separation, the proteins were transferred to a PVDF membrane, blocked with protein-free fast-blocking solution (PS108, Epizyme, China) for 30 min to prevent non-specific binding, and washed three times with 1 × Tris-buffered saline containing Tween 20 (1 × TBST). The membrane was then incubated with rabbit anti-GAPDH (5174S, CST, USA), anti-Col1(α1) (bs-10423R, Bioss Antibodies, USA), anti-Col3(α1) (bs-0548R, Bioss Antibodies), or anti-α-SMA (19245S, CST, USA) antibodies overnight at 4°C, followed by three 1×TBST washes. Then, the membrane was incubated with secondary antibodies (S0B4002, Starter, China) at room temperature for 1.5 h, followed by three 1 × TBST washes. Immunoreactive bands were detected using a ChemiDoc MP Imaging System (Bio-Rad, USA) and visualized with Tanon High-sig ECL Western Blotting Substrate (SQ201, Epizyme, China). The quantification and analysis were performed using ImageJ software to assess the protein expression levels. Antibody information is listed in **Table 2**.

**Table 2:** Information for immunofluorescence antibody and other reagents.

Reagent	Manufacturer	Cat.No.	Genera	Dilution ratio
GAPDH (D16H11) XP^®^ Rabblit mAb	Cell Signaling Technology	5174S	Rabbit	1:1000
alpha-Smooth Muscle Actin (D4K9N)XP^®^ Rabbit mAb	Cell Signaling Technology	19245S	Rabbit	1:1000
Rabbit Anti-Collagen I antibody	bioss	bs-10423R	Rabbit	1:1000
Rabbit Anti-Collagen III antibody	bioss	bs-0549R	Rabbit	1:1000
Goat anti-rabbit IgG (H+L), HRP	STARTER	S0B4002	Goat anti-rabbit	1:5000
Flotillin-1 Antibody	Cell Signaling Technology	3253S	Rabbit	1:1000
HSP70 (D1M6J) Mouse mAb	Cell Signaling Technology	46477S	Mouse	1:1000
Rabbit Anti-Mouse IgG (HRP Conjugate)	Cell Signaling Technology	58802S	Rabbit Anti-Mouse	1:5000

### Quantitative polymerase chain reaction

Total RNA from the cell and tissue samples was extracted using the trizol method. After measuring the total RNA concentration using NanoDrop™ 2000 software, reverse transcription was conducted according to the HyperScript™ III RT SuperMix for qPCR with gDNA Remover (R202-02, EnzyArtisan, China). Reverse transcription of miR-30 was performed according to the HyperScript™ III miRNA 1st Strand cDNA Synthesis Kit (by stem-loop) (R601, EnzyArtisan, China).

All the cDNA was analyzed by quantitative polymerase chain reaction (qPCR) using a 2 × S6 Universal SYBR qPCR Mix kit (Q204-05, EnzyArtisan, China). The endogenous control was *U6* snRNA or *GAPDH*. The 2^-△△^ *^t^* method was used to calculate the fold change in the expression of all mRNAs and miRNAs. The primers used in this study are listed in **Table 3**.

**Table 3:** Primer sequences.

Genes	Primer sequences
Human GAPDH-F	AAGGTGAAGGTCGGAGTCAAC
Human GAPDH-R	GGGGTCATTGATGGCAACAATA
Mouse GAPDH-F	CATCACTGCCACCCAGAAGACTG
Mouse GAPDH-R	ATGCCAGTGAGCTTCCCGTTCAG
Human-a-SMA/ACTA2 (ID59)-F	ATGCTTCTAAAACACTTTCCTGCTC
Human-a-SMA/ACTA2 (ID59)-R	AGCTTTGGCTAGGAATGATTTGG
Mouse-a-SMA/Acta2 (ID11475)-F	CTGGTATTGTGCTGGACTCTG
Mouse-a-SMA/Acta2 (ID11475)-R	GATCTTCATGAGGTAGTCGGTG
Human-COL1A1 (ID1277)-F	CCGCTTCACCTACAGCGTCA
Human-COL1A1 (ID1277)-R	TTGTATTCAATCACTGTCTTGCCC
Mouse-COL1A1 (ID12842)-F	CCTCAGGGTATTGCTGGACAAC
Mouse-COL1A1 (ID12842)-R	TTGATCCAGAAGGACCTTGTTTG
Human-COL3A1 (ID1281)-F	TGGTCTGCAAGGAATGCCTGGA
Human-COL3A1 (ID1281)-R	TCTTTCCCTGGGACACCATCAG
Mouse-Col3a1 (ID12825)-F	GACCAAAAGGTGATGCTGGACAG
Mouse-Col3a1 (ID12825)-R	CAAGACCTCGTGCTCCAGTTAG
Human-NT5E/CD73 (ID4907)-F	CACTGGGACATTCGGGTTTTG
Human-NT5E/CD73 (ID4907)-R	CTCTTTGGAAGGTGGATTGCC
Human-RORA (ID6095)-F	CACCAGCATCAGGCTTCTTTCC
Human-RORA (ID6095)-R	GTATTGGCAGGTTTCCAGATGCG
Human-FNDC5 (ID252995)-F	AGCGAGCCTGTGCTCTTCAAGA
Human-FNDC5 (ID252995)-R	GAACAGGACCACGACGATGATC
Human-IL-10 (ID3586)-F	TTCCATTCCAAGCCTGACCAC
Human-IL-10 (ID3586)-R	GCTCCCTGGTTTCTCTTCCTAAG
Human-SIPA1L1 (ID26037)-F	GCAGATGATCGACCTCCTGAGA
Human-SIPA1L1 (ID26037)-R	GCGGTAGGTTTCAGAGCAACTC
Human-USB1 (ID79650)-F	GCCCCATGCCCAGACATATG
Human-USB1 (ID79650)-R	GCAGAACCACACTCTGGGACAG
Human-CBFA2T3 (ID863)-F	CCTGGTGAACTCGACATTGACG
Human-CBFA2T3 (ID863)-R	CCGCAGAGGGAAGTTGGTG
Human-PEX19 (ID5824)-F	CCTACTCTCCAAGGATGTGCTG
Human-PEX19 (ID5824)-R	ATGACGCTGTGCTGCTCCTGAT
Human-SDK1 (ID221935)-F	GGAGTCTGTGACCCTGGACAAC
Human-SDK1 (ID221935)-R	AGAAGGTGCTCTTGACATTGAGG
Human-AAK1 (ID22848)-F	CATTTGGGCTCTTGGATGTTTG
Human-AAK1 (ID22848)-R	TTCCATCACAAATTGCCACCTG
Human-GNG5 (ID2787)-F	AGCTGCAGACTTGAAACAGTTCTG
Human-GNG5 (ID2787)-R	GGGTCTGAAGGGATTTGTACTTG
Human-TENT4B (ID64282)-F	TATCGAAGATCCTTTACAACCAGG
Human-TENT4B (ID64282)-R	ACAACGTAGGCATAATCAAAGGC
Human-ATP1A3 (ID478)-F	CATCGAGATTGAGCACTTCATCC
Human-ATP1A3 (ID478)-R	CCAGGTGTATCCGAGAATGAGG
Human-HNRNPU (ID3192)-F	GAGATTGCTGCCCGAAAGAAGC
Human-HNRNPU (ID3192)-R	TTCGCTGGAAGCCTGCAAACAG
Human-BTBD9 (ID114781)-F	CGAGATAGCCGGTCTTACTCATACT
Human-BTBD9 (ID114781)-R	TTCTGCCAAGAACGACACAGATA
Human-SMAP2 (ID64744)-F	AACCTCGACCAGTGGACTCAAG
Human-SMAP2 (ID64744)-R	ATCTGAGGTCGCCGAAAGGTCT
Human-SMUG1 (ID23583)-F	GCATGAACCCTGGACCTTTTGG
Human-SMUG1 (ID23583)-R	CTGGTCGTTTAGGATGCTCTTGG
Human-ARHGAP31 (ID57514)-F	TTCATGGATCAGAGAGCGGAGG
Human-ARHGAP31 (ID57514)-R	GCATCCTCAAGACCTGCCTTCT
Human-ARF3 (ID377)-F	GTGGTCGACAGCAATGATCGG
Human-ARF3 (ID377)-R	AGGAGTACAGCATCCCGGAGC
Human-HDGFL3 (ID50810)-F	GCAAGCAGTGAGGAAGAAGGTG
Human-HDGFL3 (ID50810)-R	TCATCTCCTGGAGATTTCCGGG
Human-ZC4H2 (ID55906)-F	CAAGCAGGAGATGGACCTTCTG
Human-ZC4H2 (ID55906)-R	GTAGACTCTAGCAGCTTGTTTAGG
Human-KBTBD8 (ID84541)-F	TTGGACGACTGTTTGCGCGATG
Human-KBTBD8 (ID84541)-R	CCTGGATTCTAGGCACAGTCATG
Human-TNRC6B (ID23112)-F	AAACATTGACCCTGAATCTGACC
Human-TNRC6B (ID23112)-R	TCTACAATGGGAGATGTGGCTG
Human-GAP43 (ID2596)-F	GAGCAGCCAAGCTGAAGAGAAC
Human-GAP43 (ID2596)-R	GCCATTTCTTAGAGTTCAGGCATG
Human-ERGIC1 (ID57222)-F	GACATTCAGGATGAGATGGGCAG
Human-ERGIC1 (ID57222)-R	CTGCCCCATTGTTCAGCGG
Human-DCAF10 (ID79269)-F	ATACAGAAGATGGGTGTCCACATA
Human-DCAF10 (ID79269)-R	GAGATATCCAGAGGACGTTGAAA
Human-IKZF4 (ID64375)-F	CTCCTGGAAAAGGACGACAGC
Human-IKZF4 (ID64375)-R	CTGGCCCACTCCCATCACAG
Human-PTGFRN (ID5738)-F	CTCCTACAGGTGTATCGTCAGC
Human-PTGFRN (ID5738)-R	AGCCACAGACACATTCTTGGGC
Human-ZBTB34 (ID403341)-F	GCCAGCTTTCTTCAGATGCAGTG
Human-ZBTB34 (ID403341)-R	CTCTTCAGCACCGACGGTAACA
Human-CLCN6 (ID1185)-F	AGTCTGCCATCCTCCAGCTCTT
Human-CLCN6 (ID1185)-R	GCCACTTGGAACAGAAATGCCG
Human-CNPPD1 (ID27013)-F	CCAGACTACTTGCAGCATGTGTC
Human-CNPPD1 (ID27013)-R	CCATTCGTCGTTGAAGACCTCC
Human-ZKSCAN8 (ID7745)-F	CGAGAGGAGTGGCTTCTTGATC
Human-ZKSCAN8 (ID7745)-R	TCCCTGTTCTCACTCCTGGTCT
Human-KAZN (ID23254)-F	CCAGAGCCAAAGAAGCCTTGCA
Human-KAZN (ID23254)-R	CTCTCCAGTGTGGCATACAGCT
Human-PPP2R5D (ID5528)-F	TACGAGACGGAGCATCACAACG
Human-PPP2R5D (ID5528)-R	GGAAGTAGGACACGGATGAGGA
Human-PHF21A (ID51317)-F	GAGACCACATTCACTTTCCCTGC
Human-PHF21A (ID51317)-R	GTGTCGCACATCAGTAACTGGC
Human-NDNF (ID79625)-F	TGATGCGCCTTTGGAGTGGAAG
Human-NDNF (ID79625)-R	GGAGAATAACTCAGTGCCTTCCT
Human-ELMO1 (ID9844)-F	CCACGACAGATCCTTTGAGGAG
Human-ELMO1 (ID9844)-R	CTCATAACCTGCTCCTTCACCAC
Human-SNX18 (ID112574)-F	GGAGGACTTCATCTCTAAGCGC
Human-SNX18 (ID112574)-R	CTTCCAGGCTTTCTCGTCGGTG
Human-NMNAT2 (ID23057)-F	GTAGTGACCTGCTGGAGTCCTT
Human-NMNAT2 (ID23057)-R	ATGATTCGGTCTGTGTCGGCTG
Human-KCNC4 (ID3749)-F	GCTCCACCACTCGAGACAGAAA
Human-KCNC4 (ID3749)-R	TACCATCGGCGCAGGCATAG
Human-COPS7B (ID64708)-F	CGTGTTGCTGAAAGACCTGGAG
Human-COPS7B (ID64708)-R	GCCAATGCAGAAATCCACTTC
Human-GDPD4 (ID220032)-F	TATTTGATCTTCATCGCCCTCC
Human-GDPD4 (ID220032)-R	GAGGCAAGGATCACGCTTACTAC

### Determination of hydroxyproline content

We accurately weighed 30–100 mg of liver tissue using the hydroxyproline assay kit (A030-2-1, Nanjing Jiancheng Bioengineering Institute, China).

### Statistical analysis

Statistical analysis was conducted using GraphPad Prism 9.0. Student’s *t*-tests were applied to assess differences between two groups. One-way analysis of variance (ANOVA), followed by Tukey’s post-tests, was used for three or more groups. Data were given as mean ± standard deviation and were shown as error bars for all experiments. Differences were considered significant at *P* < 0.05. The reported data were obtained from at least three biological replicates.

## Results

### Activation of HSCs by miRNA-30 derived from *S. japonicum* egg exosomes

**Figure 1:**
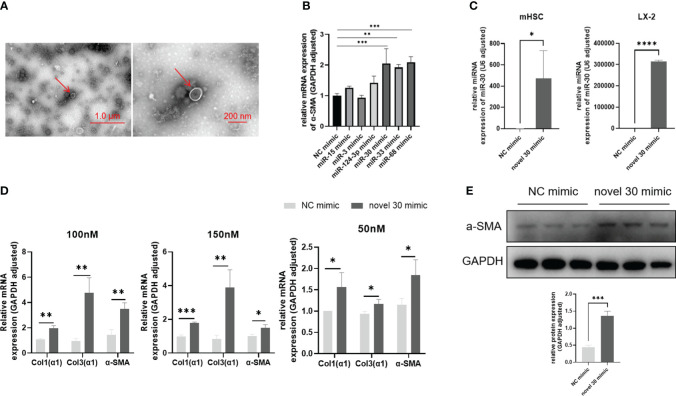
miRNA-30 derived from *S. japonicum* egg exosomes activates HSCs. **(A)** Exosomes were observed under transmission electron microscopy (TEM). **(B)** LX-2 cells were transfected with miRNA mimics for 48 h, followed by qPCR analysis to measure the mRNA levels of *α-SMA*. **(C)** LX-2 and mHSC cells were transfected with 50 nM of both NC mimic and miRNA-30 mimic, for 24 (h) Relative transcript levels of miRNA-30 were determined by qPCR. **(D)** LX-2 and mHSC cells were transfected with varying doses of NC mimic and miR-30 mimic for 48 h, followed by qPCR to quantify the mRNA levels of *α-SMA*, *Col1(α1)*, and *Col3(α1)*. **(E)** LX-2 cells were exposed to a 100 nM of both NC mimic and miRNA-30 mimic for 48 (h) The LX-2 cells were then subjected to Western blot analysis to detect their *α-SMA* protein expression levels. Both mRNA and protein levels of *α-SMA*, *Col1(α1)*, and *Col3(α1)* were normalized against *GAPDH*. *GAPDH*, 37 kDa; *α-SMA*, 43 kDa. NC, negative control; 30 mimic, miRNA-30 mimic. The results are averaged from three independent experiments. Student’s *t*-tests were carried out to assess differences between the two groups (*P < 0.05; **P < 0.01; ***P < 0.001; ****P < 0.0001).

As shown in (**Figure 1A**), we first identified egg-derived exosomes using transmission electron microscopy (TEM) and then extracted them through overspeed centrifugation. Next, we performed high-throughput sequencing and obtained a large number of miRNA sequences. From this pool of miRNA sequences, we identified six sequences associated with *S. japonicum* infection. We synthesized miRNA mimics for enhanced cellular miRNA enrichment in subsequent experimentations. These mimics were transfected into the LX-2 cell line. After 48 h, the total RNA was extracted to assess changes in the *α-SMA* levels—a hallmark of hepatic fibrosis. Based on the results obtained, we identified three types of novel miRNAs, including miRNA-33 (33) previously reported to promote HSC activation, capable of significantly up-regulating the *α-SMA* transcriptomic scales (**Figure 1B**). In contrast, other miRNAs, such as miRNA-15, miRNA-3, and miRNA-124-3p, had negligible effects on LX-2 cells. The other two novel miRNAs, miRNA-30 and miRNA-68, increased the transcription of *α-SMA* in LX-2 cells, suggesting their potential involvement in *S. japonicum* infection-induced hepatic fibrosis. In the subsequent investigations, we probed the regulatory role of miRNA-30 in *S. japonicum*-induced hepatic fibrosis.

We initially assessed the transfection efficiency and cellular tolerance of miRNA-30 mimic. First, the LX-2 cells and mHSCs were transfected with an NC mimic and miRNA-30 mimic, for 24 h. Following the transfection, total RNA was extracted from both cell lines, and miRNA-30 levels were quantified via qPCR using the stem-loop method. The results demonstrated successful transfection and sustained presence of miRNA mimics within the HSCs of different species (human and mouse) (**Figure 1C**). Subsequently, we determined the optimal concentration of miRNA-30 mimic in both cell types. The LX-2 cells tolerated up to 150 nM of the miRNA mimics (**Figure 1D**), whereas >50 nM of miRNA mimics induced cell death in mHSCs (**Figure 1D**). Hence, we employed 50 nM of the miRNA-30 mimic for subsequent experimentations with mHSCs.

To further elucidate the activating influence of miRNA-30 on HSCs, we re-transfected the LX-2 cells with 100 and 150 nM of the NC mimic and miRNA-30 mimic. Total RNA was extracted after 48 h of transfection, and alterations in the hepatic fibrotic factor levels were assessed through qPCR. Our findings revealed that both 100 and 150 nM concentrations of miRNA-30 mimic led to an up-regulation of *Col1(α1)*, *Col3(α1)*, and *α-SMA* at the mRNA level (**Figure 1D**). Next, we conducted a Western blot analysis of the total protein extracted from the LX-2 cells transfected with 100 nM of both the NC mimic and miRNA-30 mimic to ascertain the upregulation of *α-SMA* at the protein level (**Figure 1E**). After establishing the ability of miRNA-30 to promote LX-2 activation, we extended our investigation to mHSCs. Remarkably, treatment with 50 nM of miRNA-30 mimic also resulted in the upregulation of *Col1(α1)*, *Col3(α1)*, and *α-SMA* at the mRNA level (**Figure 1D**).

### Validation of the target genes of miRNA-30

**Figure 2:**
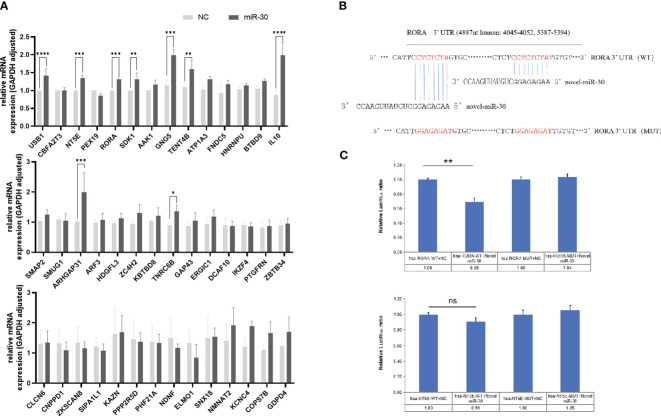
MiRNA-30 of *S. japonicum* egg exosome promotes the progression of hepatic fibrosis by targeting the host RORA gene in HSCs. **(A)** LX-2 cells were transfected with 150 nM of NC and miR-30 mimics for 48 (h) The mRNA expression of the first 42 candidate genes was measured by qPCR. **(B)** Sequence alignment was performed between miR-30 and the 3’-UTR of candidate gene *RORA*, which revealed two binding sites. **(C)** Validation of genes *RORA* and *NT5E* dual-luciferase target. The mRNA levels of 42 potential targets were normalized to *GAPDH*. NC, negative control; 30 mimic, miRNA-30 mimic. The results are averaged from three independent experiments. Student’s *t*-tests were employed to assess differences between the two groups (*P < 0.05; **P < 0.01; ***P < 0.001; ****P < 0.0001) ns, no significance.

MiRNA-30 target genes were predicted using the MicroRNA Target Prediction Database (miRDB), which revealed numerous potential target genes in both human and mouse models. The results showed 624 genes scored above 50 in humans and 651 in mice. Common to human and mouse, the top-scoring 42 target genes were validated by qPCR following the transfection of LX-2 cells with the NC and miRNA-30 mimics for 48 h *in vitro*. Among these, the mRNA levels of nine genes, including *USB1*, *NT5E*, *RORA*, *SDK1*, and *IL10*, were significantly up-regulated (**Figure 2A**), suggesting their involvement in the miRNA-30-mediated regulation of host hepatic fibrosis.

Based on the prediction software scores, the retinoic acid receptor (RAR)-related orphan receptor alpha (*RORA*) and 5’-nucleotidase ecto (*NT5E*) genes were selected for further investigations. To verify whether *RORA* and *NT5E* are the direct target genes of miRNA-30, we cloned the 2 target genes 3’-UTR-indicated sequence into the pmiR-GLO dual luciferase reporter vector, respectively. As shown in (**Figure 2B**), two *RORA* gene sites are complementary to eight bases of miRNA-30 sequence. Co-transfection of tool cells (293T cells) with vectors constructed from both *RORA* and *NT5E* genes and miRNA-30 mimics or NC significantly reduced the luciferase activity in the cells co-transfected with the *RORA* WT vector and miRNA-30 mimics compared to that of the NC-treated cells (**Figure 2C**). Conversely, mutation of the seed-binding sequence in the 3’-UTR of the *RORA* gene removed this inhibition (**Figure 2C**). While binding of the *NT5E* gene to miRNA-30 decreased the luciferase activity, it did not reach significance (**Figure 2C**), suggesting an interaction between miRNA-30 and the 3’-UTR of LX-2 target gene *RORA* mRNA.

### Promotion of hepatic fibrosis in mice by *S. japonicum* egg exosome-derived miRNA-30

To further investigate whether miRNA-30 could also contribute to the progression of hepatic fibrosis in mice, we conducted *in vivo* overexpression experiments by injecting rAAV8-miR-30 into the tail veins of healthy female C57BL/6 mice. Subsequently, we monitored dynamic changes in the hepatic fibrosis-related indicators.

**Figure 3:**
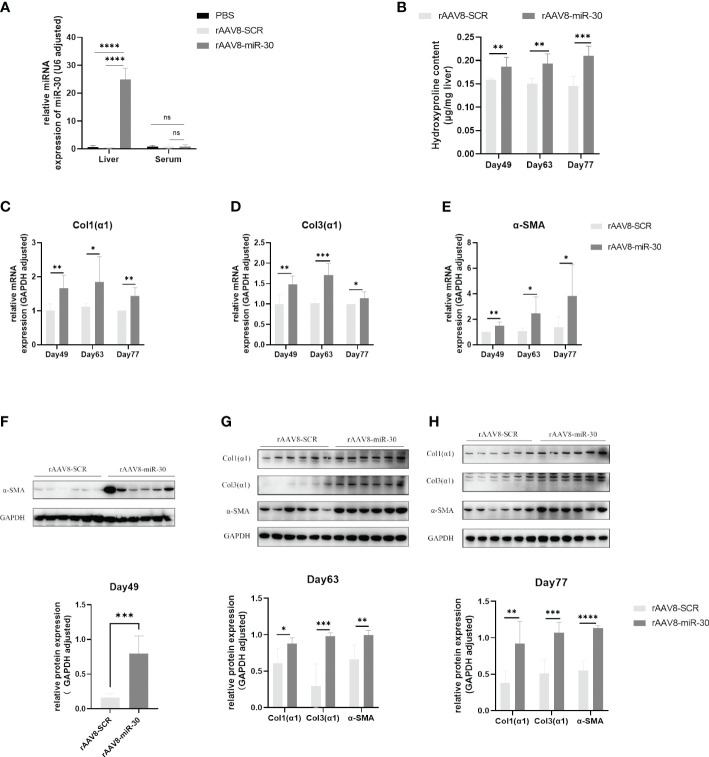
Overexpression of miRNA-30 induces hepatic fibrosis in naive mice. Healthy six-week-old female C57BL/6 mice were injected with of PBS, rAAV8-SCR (blank vector), and rAAV8-miR-30 (overexpression vector) into the tail vein, with a viral titer of 5 × 10 (11) or 1 × 10 (12) vector genomes per mouse. **(A)** Liver and serum samples were collected three weeks post-injection to measure the miRNA-30 expression through qPCR, with normalization to *U6* RNA (*n* = 6). **(B)** Liver tissues harvested on days 49, 63, and 77 post-injection were analyzed for hydroxyproline content (*n* = 6). **(C–E)** qPCR to quantify the mRNA levels of *α-SMA*, *Col1(α1)*, and *Col3(α1)* (*n* = 6). **(F–H)** Western blot to quantify the protein levels of *α-SMA*, *Col1(α1)*, and *Col3(α1)* (*n* = 6). Both mRNA and protein levels of *α-SMA*, *Col1(α1)*, and *Col3(α1)* were normalized against *GAPDH*. *GAPDH*, 37 kDa; *α-SMA*, 43 kDa; *Col1(α1)*, 130 kDa; *Col3(α1)*, 117 kDa. rAAV8, recombinant adeno-associated virus serotype 8; SCR, scrambled. Statistical significance between two or more groups was evaluated using Student’s *t*-test and one-way ANOVA (*P < 0.05; **P < 0.01; ***P < 0.001; ****P < 0.0001).

Upon entry into the cell, recombinant adeno-associated virus (rAAV) can persist as additional DNA, exhibiting a stable presence within the cell. Typically, the gene expression peaks around weeks 2–3 post-entry and can endure for weeks to months. We injected the rAAV8-miR-30, rAAV8-SCR NC, and PBS blank control into the tail vein of C57BL/6 mice at a dosage of 5×10^11^ viral genomes per mouse. We obtained the liver and serum samples from the mice three weeks after the injection. Stem-loop reverse transcription of miRNA-30 followed by qPCR analysis showed that the sera of all the three groups (rAAV8-miR-30, rAAV8-SCR NC, and PBS blank control) did not exhibit overexpression of miRNA-30 (**Figure 3A**). Conversely, a marked upregulation of miRNA-30 was observed in the livers of the mice injected with rAAV8-miR-30 (**Figure 3A**), affirming the accurate targeting of the liver and processing of miRNA-30 within the livers of the mice. Additionally, the livers of the mice injected with the rAAV8-SCR NC exhibited no discernible variance compared to that shown by the PBS group, indicating that the recombinant adeno-associated viral empty vector did not exert any additional influence on the processing and maturation of miRNA-30 (**Figure 3A**). Consequently, the rAAV8-miR-30 overexpression group and the rAAV8-SCR NC were employed for the subsequent experiments.

We increased the viral titer to 1×10^12^ viral genomes per mouse and injected it into the tail vein of the C57BL/6 mice. The mice were euthanized and their liver tissue was collected on days 49, 63, and 77 post-injection to assess hepatic fibrosis-related markers. Our findings revealed a significant increase in the hydroxyproline content in the rAAV8-miR-30 overexpression group compared to that in the rAAV8-SCR NC group at all three time points (**Figure 3B**). Furthermore, the qPCR analysis of *Col1(α1)*, *Col3(α1)*, and *α-SMA* mRNA levels in the rAAV8-miR-30-induced mouse liver tissues demonstrated a substantial exacerbation of fibrosis at all three time points compared to that observed in the rAAV8-SCR NC group (**Figures 3C–E**). The Western blot results also corroborated this trend (**Figures 3F–H**). A comprehensive analysis of the hydroxyproline content and hepatic fibrosis factors (*Col1(α1)*, *Col3(α1)*, and *α-SMA*) at both the mRNA and protein levels over time revealed a progressive aggravation of hepatic fibrosis in mice following the *in vivo* overexpression of miRNA-30.

### Inhibition of miRNA-30 obtained from *S. japonicum* egg-derived exosomes delays the progression of schistosomiasis-induced hepatic fibrosis in mice

After confirming the pro-fibrotic role of miRNA-30 in the WT female C57BL/6 mice, we hypothesized that inhibiting miRNA-30 could be a therapeutically promising option for schistosomiasis-induced hepatic fibrosis. To delve deeper into its involvement in *S. japonicum*-induced hepatic fibrosis, we administered rAAV8-anti-miR-30 to female C57BL/6 mice infected with *S. japonicum*. This *in vivo* approach aimed to silence miRNA-30 and assess its impact on the regulatory and therapeutic aspects of hepatic fibrosis in schistosomiasis. We employed the rAAV8-anti-miR-30 sponge (sponge vector) as the inhibitor. This sponge vector binds to miR-30 and inhibits its function. The potency and specificity of the sponge vector were validated in 293T cell lines.

**Figure 4:**
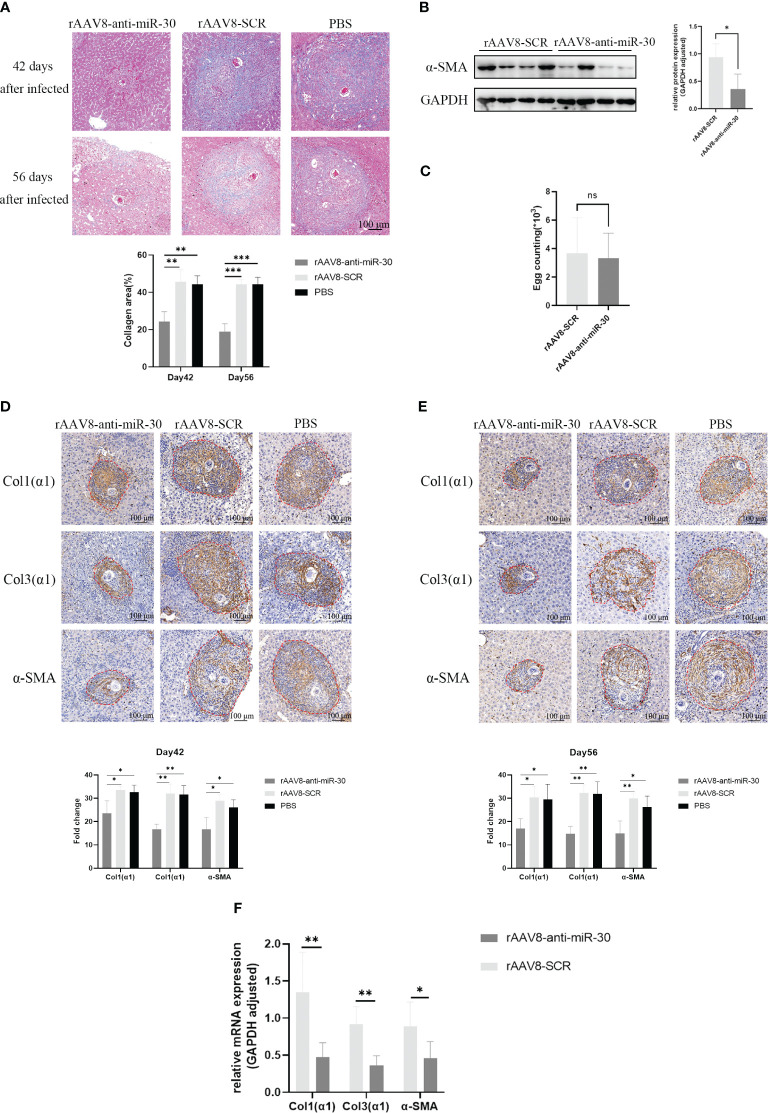
Inhibition of miRNA-30 from *S. japonicum* egg-derived exosomes delays the progression of hepatic fibrosis in schistosomiasis in mice. Mice received an injection of rAAV8-SCR or rAAV8-anti-miR-30-sponge vectors at a dose of 1 × 10 (12) virus genomes or PBS via the tail vein. After 3 weeks, these mice were dermally exposed to 20 ± 1 *S. japonicum* cercariae. Liver samples were collected at days 42 and 56 post-infection. **(A)** Granulomas and collagen deposition were assessed through Masson’s trichrome staining. **(B)** Expression of *α-SMA* at the protein level was examined on day 42 post-infection through Western blot analysis. **(C)** Egg count in mouse liver tissue showing there was no statistical difference between the two groups (*n* = 6). **(D, E)** Immunohistochemical analysis on days 42 and 56 post-*S. japonicum* infection revealed positively stained areas for *Col1(α1)*, *Col3(α1)*, and *α-SMA*. **(F)** qPCR to quantify the mRNA levels of *α-SMA*, *Col1(α1)*, and *Col3(α1)* (*n* = 6). All images were magnified 10-fold. Quantification of *α-SMA* protein levels was normalized to *GAPDH*. *GAPDH*, 37 kDa; *α-SMA*, 43 kDa. rAAV8, recombinant adeno-associated virus serotype 8; SCR, scrambled. Statistical significance between two or more groups was evaluated using Student’s *t*-test and one-way ANOVA (*P < 0.05; **P < 0.01; ***P < 0.001. ns, no significance.

We injected rAAV8-anti-miR-30 and rAAV8-SCR into the tail vein of 6-week-old healthy female C57BL/6 mice, while simultaneously exposing them percutaneously to 20 ± 1 *S. japonicum* cercariae. The liver tissues of these infected mice were then collected for immunohistochemistry, Western blot, and Masson trichrome staining analyses on days 42 and 56 post-infection. Masson trichrome staining demonstrated a notable decrease in the hepatic collagen areas and granuloma size in the mice injected with the rAAV8-anti-miR-30 sponge vector compared to those injected with the SCR control vector (**Figure 4A**). Western blotting results showed the down-regulation of *α-SMA* at the protein level (**Figure 4B**), while the number of *S. japonicum* eggs was not statistically different between the two groups (**Figure 4C**). Immunohistochemical analysis revealed significant reductions in the areas of *Col1(α1)*, *Col3(α1)*, and *α-SMA* on days 42 and 56 post-infection in the rAAV8-anti-miR-30-treated group compared to that in both the NC and PBS groups (**Figures 4D, E**). Moreover, qPCR results also showed the same trend (**Figure 4F**).

## Discussion

HSCs, constituting approximately 8–10% of total liver cells, are the precursor of principal fibroblasts in the liver (34). Their activation is crucial for initiating and advancing hepatic fibrosis, as they are responsible for the reduced degradation of the ECM and pathological deposition (35). Therefore, it is imperative to understand HSC activation. Various stimuli trigger HSC activation under pathological conditions, such as physical, chemical, viral, or biological insults, causing their transition into myofibroblasts characterized by *α-SMA* expression and ECM synthesis (36–38). Exogenous agents, such as exosomes or miRNAs, can also induce HSC activation. In our previous work, we have discussed the critical regulatory role of miRNAs in hepatic fibrosis (39). Herein, we investigate the activating effect of miRNA-30 mimics on HSCs via transfection. While higher doses of miRNA mimics (>50 nM) induced cell death in mHSCs, possibly because of the more demanding isolation and culture requirements of primary mHSCs, LX-2 cells could tolerate increased transfection doses. The subsequent analysis revealed significant upregulation of *Col1(α1)*, *Col3(α1)*, and *α-SMA* at both the mRNA and protein levels, indicating enhanced expression of fibrosis-associated factors in response to miRNA-30. These findings demonstrate that miRNA-30 derived from *S. japonicum* exosomes activates both human and mouse HSCs, promoting the expression of fibrosis-associated factors. Hence, we hypothesize that miRNA-30 exhibits cross-species activation potential.

In contrast to the conventional negative regulation of target genes by miRNAs (40), our study revealed that the *S. japonicum* egg exosome-derived miRNA-30 can trigger the activation of host HSCs by up-regulating its target gene, *RORA*, within host cells, which accelerates the fibrotic process. While miRNA-30 does not participate in the degradation or repression of target genes, its role in enhancing the stability of target genes or their translational processes cannot be overlooked. This phenomenon can be attributed to miRNAs regulating target genes or cell cycle arrest, where microRNPs toggle between the repression and activation of translation (41). In addition, miRNAs have been documented to boost the transcriptional and translational stability of mRNAs in viruses and eukaryotes alike (42). Thus, our findings offer insights into the potential positive regulation of host target genes by miRNAs across species.

*RORA*, an orphan receptor, is known to have a role in the regulation of various physiological processes, including metabolism, cellular immunity, inflammatory responses, and circadian rhythms (43, 44). This receptor operates across several human organs, such as the brain, heart, liver, testis, and skin (45). The *RORA* gene yields four isoforms through selective promoter and exon splicing: *RORa1*, *RORa2*, *RORa3*, and *RORa4* (44). While these isoforms share common DNA and putative ligand-binding domains, they differ in their amino-terminal sequences, leading to slight variations in DNA-binding preferences (43, 46). Notably, *RORa4* is the predominant isoform of hepatic nuclear receptor RORA. Its transcriptional activity can be modulated by various external stimuli in the HepG2 human hepatocellular carcinoma cells, thereby influencing disease progression (47). We propose that miRNA-30 derived from *S. japonicum* egg exosomes may enhance the transcriptional stability of *RORa4* by targeting it in host HSCs, thereby promoting hepatic fibrosis under *S. japonica* infection. However, further investigation is warranted to elucidate the specific pro-fibrotic mechanisms involved.

The progression of hepatic pathology under *S. japonicum* infection is a multifaceted process, primarily characterized by the formation of granuloma and subsequent hepatic fibrosis. Apart from SEA, exosomes, their secreted proteins, and RNAs also play critical roles in hepatic fibrosis process (31, 48). All these events involve an intricate dysregulation of gene expression, including miRNAs, within the infected liver tissues. In a previous study, we showed that the miRNA-30 originating from *S. japonicum* egg exosomes can activate HSCs, the principal effector cells in hepatic fibrosis, in both human and murine models *in vitro*. Herein, our study validates the capability of *S. japonicum* egg exosomes to activate HSCs, which in turn influences hepatic fibrosis. The findings reveal that the overexpression of miRNA-30, which is exacerbated over time, substantially increases the number of fibrosis markers in the livers of healthy mice. Hence, the miRNA-30 derived from *S. japonicum* egg exosomes can induce hepatic fibrosis in mice. Furthermore, inhibition of miRNA-30 in infected mice using the rAAV8-anti-miR-30 sponge vector led to a reduction in both the granuloma area and the collagenous area surrounding liver granulomas, which delayed the progression of hepatic fibrosis. We have previously shown that, similar to miRNA-33, miRNA-30 also promotes hepatic fibrosis across species (39). In addition, miRNA-68 (Section 3.1) is capable of activating HSCs *in vitro*. Hence, the role of miRNA-68 in schistosomiasis-induced hepatic fibrosis should be further investigated.

Understanding the mechanisms underlying hepatic fibrosis has long been a primary focus of schistosomiasis research. Hepatic fibrosis in schistosomiasis is characterized as an immunopathologic condition. SEA, a key regulator in the progression of schistosomiasis, can induce a type-2 immune response. Furthermore, pro-fibrotic cytokines, such as *IL-13* and *TGF-β1*, released during the immune response, can activate HSCs and drive hepatic fibrosis via the SMAD pathway (9). The study findings indicate that miRNA-30, specific to *S. japonicum* egg exosomes, promotes hepatic fibrosis in schistosomiasis across species.

To conclude, this study showed that in parasitic infections such as those by *Schistosoma* spp., certain miRNAs exhibit pro-fibrotic properties, while others act as inhibitors, regardless of whether they originate from the host or the parasite. The involvement of miRNAs in regulating hepatic fibrosis, coupled with their stable and easily detectable nature, offers novel avenues for monitoring and treating fibrotic liver diseases. This study sheds light on the pro-fibrotic role of miRNA-30 in schistosomiasis and enhances our understanding of how parasite-derived miRNAs regulate host pathology. It identifies potential targets that intervene in the treatment of schistosomiasis-induced hepatic fibrosis. In addition, it offers novel insights into the pathomechanisms responsible for hepatic fibrosis resulting from *S. japonicum* infection.

## Data availability statement

The original contributions presented in the study are included in the article/**Supplementary Material**. Further inquiries can be directed to the corresponding authors.

## Ethics statement

The animal study was approved by the Laboratory Animal Welfare & Ethics Committee (LAWEC) of the National Institute of Parasitic Diseases, Chinese Center for Disease Control and Prevention (Chinese Center for Tropical Diseases Research). The study was conducted in accordance with the local legislation and institutional requirements.

## Author contributions

YC: Conceptualization, Formal analysis, Methodology, Writing – original draft, Writing – review & editing. YH: Formal analysis, Methodology, Writing – review & editing. HZ: Formal analysis, Methodology, Writing – original draft. NJ: Methodology, Writing – original draft. YW: Methodology, Writing – original draft. JZ: Methodology, Writing – original draft. YS: Formal analysis, Supervision, Writing – review & editing. GY: Supervision, Writing – review & editing. JC: Conceptualization, Formal analysis, Funding acquisition, Supervision, Writing – original draft, Writing – review & editing.

## Supplementary material

Supplementary Data Sheet 1 Excel spreadsheet containing, in separate sheets, the numerical data for **Figures 1B–E**, **2A**, **3A–H**, **4B, C, F**.

Supplementary Data Sheet 2 Sourced data for the immunoblotting, TEM, masson staining, immunofluorescence, and sequence alignment are shown in **Figures 1A, E**, **2B, C**, **3F–H**, **4A, B, D–F**.
