# Supplementary figures and images for: Induction of hepatic fibrosis in mice with schistosomiasis by extracellular microRNA-30 derived from Schistosoma japonicum eggs
Source: Front Immunol. 2024 Jul 30;15:1425384. doi: 10.3389/fimmu.2024.1425384 (PMC11319242; doi:10.3389/fimmu.2024.1425384)

Figure 3F

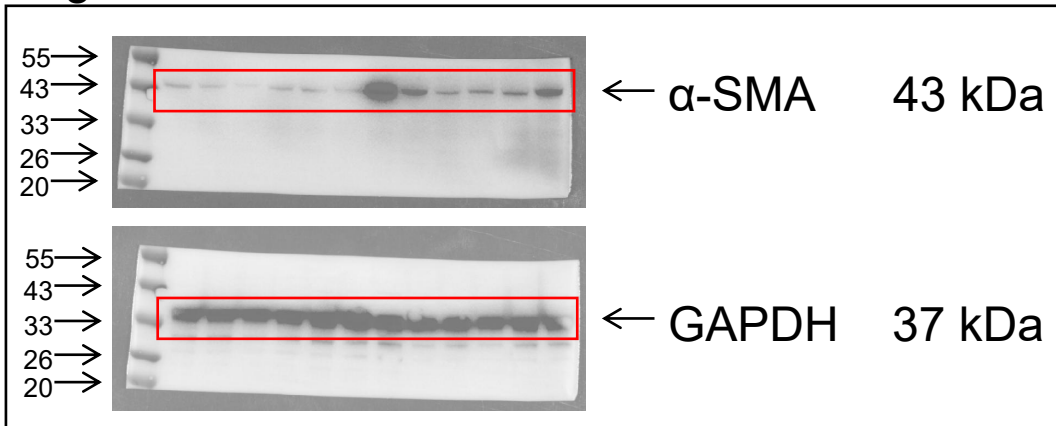

Figure 3G

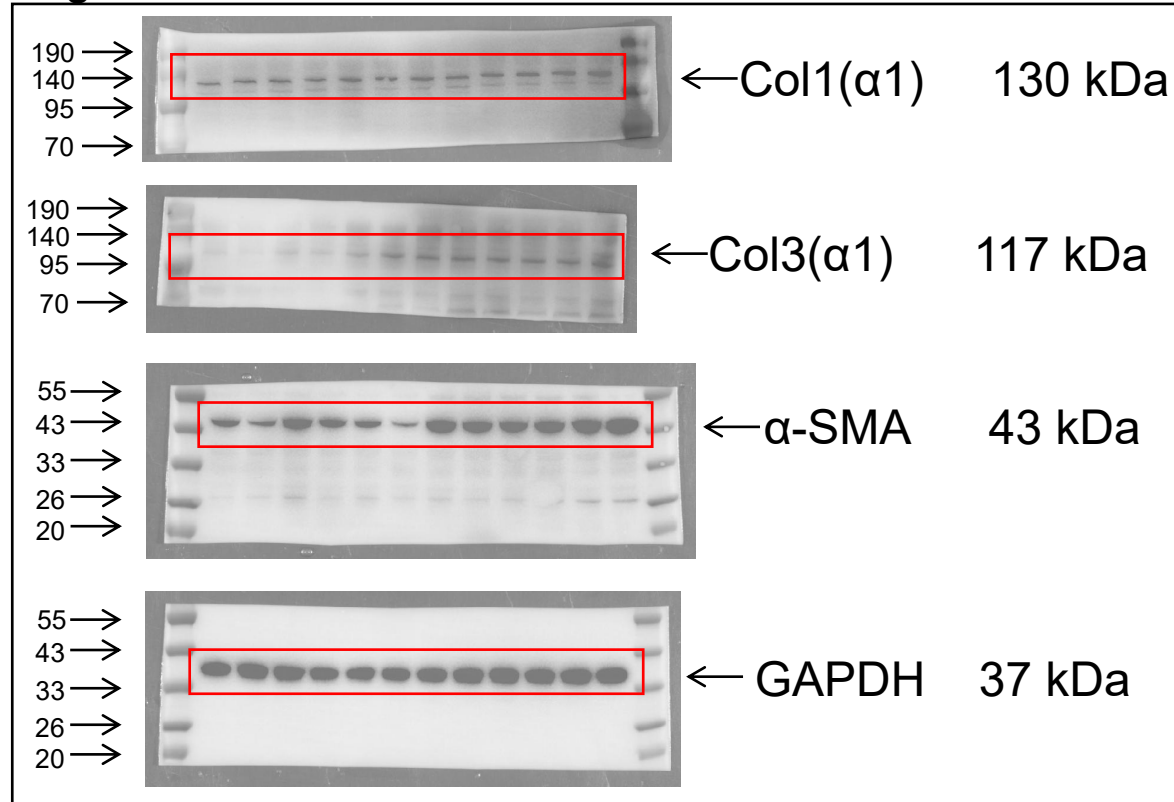

Figure 3H

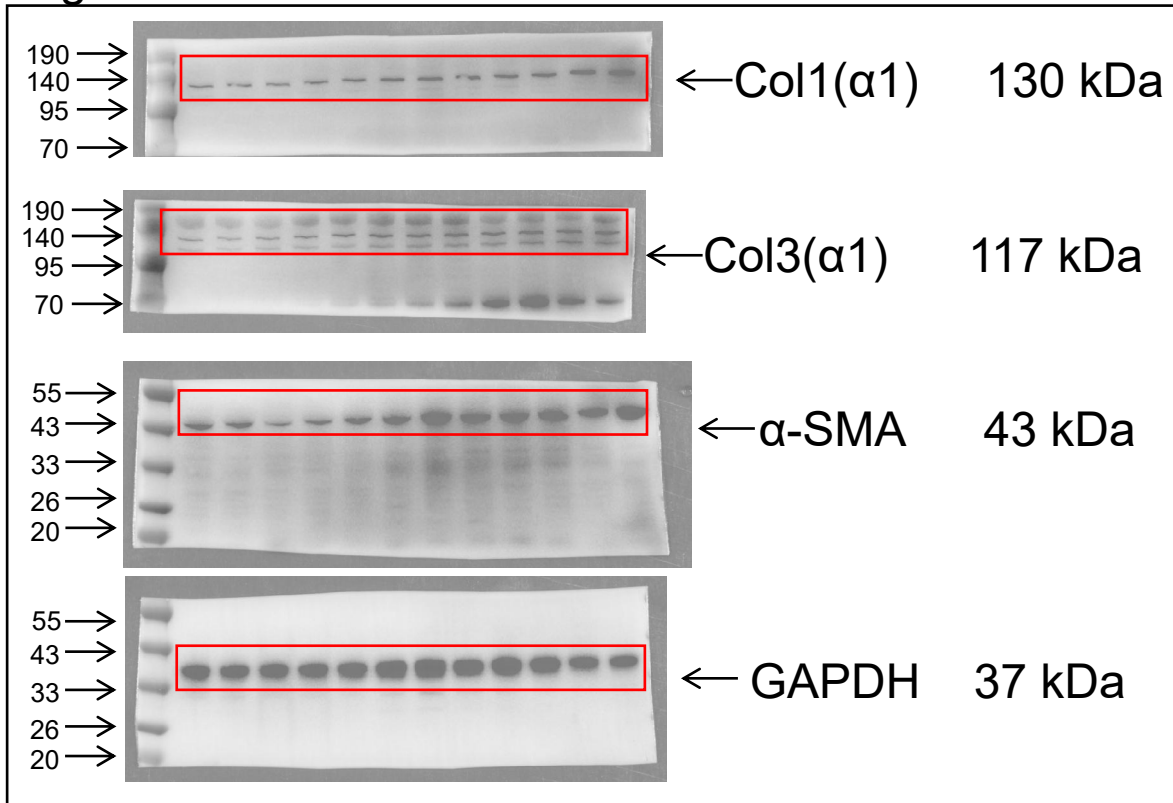

Figure 4B

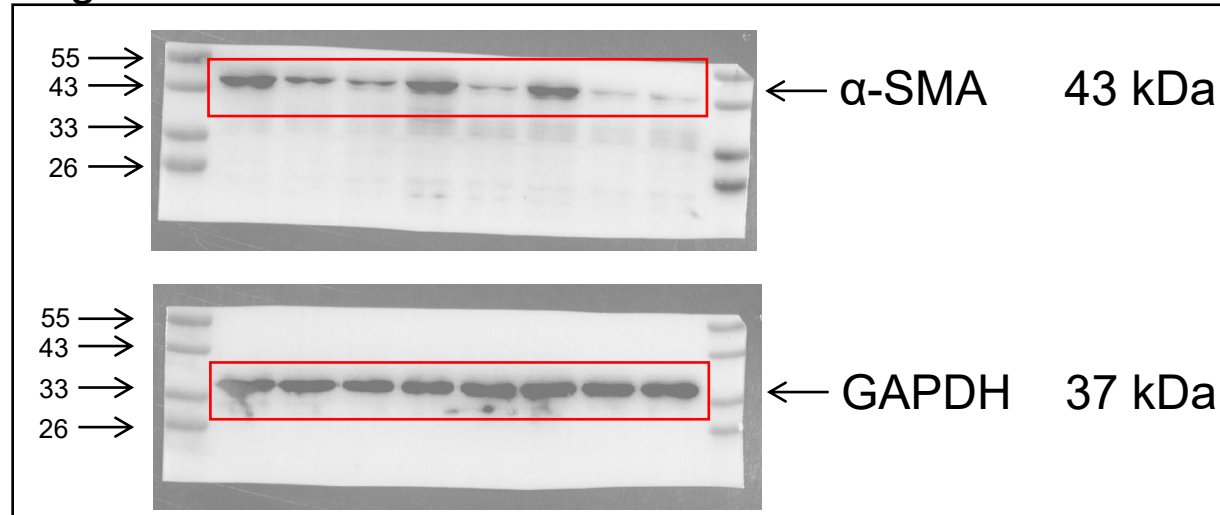

Supplement: Supplementary Data Sheet 1 — Excel spreadsheet containing, in separate sheets, the numerical data for Figures 1B–E , 2A , 3A–H , 4B, C, F . [file DataSheet_1.pdf]
